# Macro-Kinematic Differences Between Sprint and Distance Cross-Country Skiing Competitions Using the Classical Technique

**DOI:** 10.3389/fphys.2018.00570

**Published:** 2018-05-17

**Authors:** Finn Marsland, Judith Anson, Gordon Waddington, Hans-Christer Holmberg, Dale W. Chapman

**Affiliations:** ^1^UC Research Institute for Sport and Exercise, University of Canberra, Canberra, ACT, Australia; ^2^Australian Institute of Sport, Canberra, ACT, Australia; ^3^The Swedish Winter Sports Research Centre, Mid-Sweden University, Östersund, Sweden; ^4^School of Sport Sciences, UiT The Arctic University of Norway, Tromsø, Norway

**Keywords:** kinematics, cycle length, cycle rate, performance analysis, wearable sensors, Winter Olympics

## Abstract

We compare the macro-kinematics of six elite female cross-country skiers competing in 1.1-km Sprint and 10.5-km Distance classical technique events on consecutive days under similar weather and track conditions. The relative use of double pole (DP), kick-double pole (KDP), diagonal stride (DS), tucking (Tuck) and turning (Turn) sub-techniques, plus each technique’s respective velocities, cycle lengths and cycle rates were monitored using a single micro-sensor unit worn by each skier during the Sprint qualification, semi-final and finals, and multiple laps of the Distance race. Over a 1.0-km section of track common to both Sprint and Distance events, the mean race velocity, cyclical sub-technique velocities, and cycle rates were higher during the Sprint race, while Tuck and Turn velocities were similar. Velocities with KDP and DS on the common terrain were higher in the Sprint (KDP +12%, DS +23%) due to faster cycle rates (KDP +8%, DS +11%) and longer cycle lengths (KDP +5%, DS +10%), while the DP velocity was higher (+8%) with faster cycle rate (+16%) despite a shorter cycle length (-9%). During the Sprint the percentage of total distance covered using DP was greater (+15%), with less use of Tuck (-19%). Across all events and rounds, DP was the most used sub-technique in terms of distance, followed by Tuck, DS, Turn and KDP. KDP was employed relatively little, and during the Sprint by only half the participants. Tuck was the fastest sub-technique followed by Turn, DP, KDP, and DS. These findings reveal differences in the macro-kinematic characteristics and strategies utilized during Sprint and Distance events, confirm the use of higher cycle rates in the Sprint, and increase our understanding of the performance demands of cross-country skiing competition.

## Introduction

From its early beginnings in the late 1990s, the cross-country (XC) skiing sprint event (Sprint) has become a regular feature at all levels of International Ski Federation (FIS) international competition. Indeed, Sprint events (including the Team-Sprint) now constitute more than 30% of the total events on the World Cup circuit, one third of individual events at the World Junior and U23 Championships, and one third of events at the Winter Olympics and World Championships (FIS, 2017).

FIS Sprint events can be between 800 and 1800 m in length, typically taking 2 – 4 min to complete. This contrasts with traditional distance XC skiing events (Distance), which range from 5 to 30 km for women and 10 – 50 km for men at the World Championship and Winter Olympic levels, and can be as long as 90 km on the ski marathon circuit (FIS, 2018). It is thus not surprising that Sprint and Distance specialists have developed, although there remain “all-rounders” who contend for medals in both categories (Sandbakk et al., 2010; Sandbakk and Holmberg, 2014).

Over the past decade or so, several key studies have expanded our insight into Sprint performance (Zory et al., 2005; Stöggl et al., 2007; Vesterinen et al., 2009; Andersson et al., 2010; Sandbakk et al., 2011, 2012b). Examining physiological and kinematic responses during a simulated classic Sprint competition on a treadmill, Stöggl et al. (2007) concluded that performance depends not only on physiological factors such as anaerobic capacity and fatigue resistance, but also on the technique used as skiers who were able to utilize the double pole (DP) sub-technique longer performed better. This connection between choice of sub-technique and performance was confirmed by Andersson et al. (2010), who reported that during a simulated freestyle Sprint competition on snow the fastest skiers used a “higher gear” (G3 over G2 technique) to a greater extent. These XC skiing macro-kinematic variables – the relative use of each sub-technique, as well as the associated velocities, cycle lengths and cycle rates – are adapted continuously by each competitor in response to the varying terrain and conditions during a competition, within the constraints of their own strengths/weaknesses and/or personal preference (Myklebust et al., 2011; Sandbakk et al., 2011; Marsland et al., 2017).

Andersson et al.’s (2010) investigation was the first to assess macro-kinematics over the entire length of an on-snow competition. Previous kinematic analyses of this nature focussed on these parameters only for short sections of track using video analysis (Smith and Heagy, 1994; Bilodeau et al., 1996), and, more recently, force plates under the snow (Mikkola et al., 2013; Andersson et al., 2014). Velocities for different sections of a course have been reported, though without examining the relative usage of specific sub-techniques (Sandbakk et al., 2011, 2016; Bolger et al., 2015).

Recent developments in micro-sensor technology provide novel possibilities for performance analysis in the field, enabling XC skiing macro-kinematics to be monitored continuously over an entire course (Myklebust et al., 2011; Sakurai et al., 2014, 2016; Marsland et al., 2017). This technology is still developing, with different micro-sensor configurations being investigated (Stöggl et al., 2014; Rindal et al., 2017; Seeberg et al., 2017), and to date only limited full competition data have been reported. The greatest challenge in comparing events at different locations is that the topography of each course is unique, and, moreover, snow conditions even at the same location can vary considerably from day to day (Wagner and Horel, 2011). Previous work by the authors revealed that macro-kinematic strategies also vary for each individual skier (Marsland et al., 2017).

The present study was designed to compare and contrast macro-kinematic variables utilized by the same athletes under similar conditions for both Sprint and Distance competitions. By comparing data collected from the same section of track involved in both events, we sought to provide new insights into the demands of XC skiing competition. We anticipated that velocities and cycle rates would be greater during the Sprint competition than the Distance event, and that differences in cycle lengths and the relative use of each sub-technique would be apparent. Furthermore, this work would increase the limited amount of published competition data available on female skiers, and facilitate characterisation and subsequent comparison as more findings are reported.

## Materials and Methods

### Participants

Six female XC skiers participated, including two medallists at the World Cup or World Championship level (**Table 1**) and four Winter Olympians. All of these athletes volunteered to participate after being contacted via their team coach and were provided with written information about the study and given the opportunity to ask questions. Each athlete provided her written informed consent prior to participation, with ethical approval provided by the University of Canberra Committee for Ethics in Human Research and the Australian Institute of Sport Ethics Committee.

**Table 1:** Characteristics of the participants (means ± SD).

Characteristics	Values (*n* = 6)
Age (years)	24.8 ± 4.4
Body height (m)	1.66 ± 0.06
Body weight (kg)	56.7 ± 5.2
FIS Sprint rank (points)	83.9 ± 64.6
FIS Distance rank (points)	65.6 ± 45.2

### Equipment

A single micro-sensor unit (MinimaxX^TM^ S4, Catapult Innovations, Melbourne, Australia) containing a triaxial accelerometer (100 Hz, ±6 g), gyroscope (100 Hz, ±1,000 d/s) and a GPS device (Fastrax, 10 Hz) was secured to the middle of the upper back using a thin chest harness. This unit was positioned as described by Marsland et al. (2012), and calibrated according to Harding et al. (2008).

### Study Design

**FIGURE 1:**
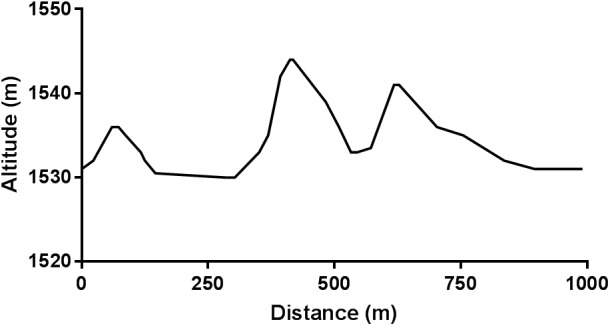
Altitude profile for the common terrain.

Data were collected during FIS Sprint and Distance competitions held on consecutive days. These race courses were designed by the organizing committee according to FIS homologation rules using the available terrain, and were approved for FIS international competition. Data were collected as the skiers covered the Sprint and Distance race courses, which included a common section of track approximately 1.0-km long. This section contained three uphill (total climb 27 m) and three downhill segments, as well as a long straight section leading into the finishing/lap area (**Figure 1**). The Sprint race was approximately 1.1-km in length (total climb 27 m), while the 10.5-km Distance event involved three laps of a loop approximate 3.5-km long (total climb per lap 85 m).

In the Sprint all skiers competed in a qualification round (where they were seeded on the basis of their FIS points, the highest ranking starting first), after which the best twelve were seeded into two semi-final rounds. The fastest two skiers from each semi-final, plus the next two fastest skiers from either semi-final, progressed through to an A-final, following the same procedure as used for FIS World Cup events. The remaining skiers from the semi-final rounds competed in a B-final race. All the participating skiers were monitored during all three rounds of racing (qualification, semi-final and A- or B-final). Ninety minutes elapsed between the start of the qualification round and start of the finals, which were completed within 45 min.

The Distance event, held the day after the Sprint competition with similar snow conditions, began with a mass start, with the highest-ranked skiers seeded at the front. The snow temperature in the stadium varied between -2° and -1°, with the air temperature warming from -2° to +2°. The courses were prepared by an experienced snow groomer using a Piston Bully machine, and the tracks were firm. All skiers used their own equipment, with ski waxing by their personal coaches, who indicated that they used the same glide wax on both days.

### Classification of Technique

Data from the micro-sensors was imported into analytical software (Makesens V70.6, Appsen, Canberra, ACT, Australia), which classified the sub-technique employed as double pole (DP), kick-double pole (KDP), diagonal stride (DS), tucking (Tuck) or turning (Turn). DP involves simultaneous pushing with both arms with no propulsion from the legs; KDP has a kick from one leg added in the middle of the DP cycle; DS involves kicking with one leg and pushing with the opposite arm in an alternating manner. All these three cyclical techniques were identified using an algorithm based on filtered gyroscope and accelerometer signals, predominantly using consecutive peaks in the Pitch gyroscope signal filtered at 1 Hz in the manner described by Marsland et al. (2015). Turn was identified using the rate of change of GPS direction. Tuck is when a skier is in an aerodynamic bent-over position, and was detected through filtered accelerometer signals. These classifications were subsequently manually checked for errors by a cross-country skiing coach with extensive experience of evaluating such micro-sensor data, using a spreadsheet (Excel 2010, Microsoft, Seattle, WA, United States) together with visual analysis of plots of the accelerometer and gyroscope values. If there was any doubt, the sub-technique was classified as miscellaneous (Misc). For each cyclical sub-technique a full cycle was defined as lasting from one pole plant to the next pole plant on the same side (Marsland et al., 2017).

### Statistical Analyses

The Wilcoxon matched-pair non-parametric test was used to compare the mean kinematic parameters associated with the Sprint and Distance events, with the mean differences (MDiff) expressed as percentages and an alpha level of *p* = 0.1 to reduce the likelihood of a type II statistical error. Macro-kinematic variables were averaged across the three Sprint rounds, and for the common terrain across the second and third laps of the Distance race (the first lap was not analyzed because of differences in the course related to the mass start). Statistical analyses were performed using GraphPad Prism (GraphPad Software, La Jolla, CA, United States) and Excel 2010 software. Unless otherwise stated, all values are presented as mean ± SD.

## Results

### Full Course

There was no statistically significant difference in the mean overall velocity of the skiers participating in the entire 1.1-km Sprint and 10.5-km Distance events, and mean finishing times across the rounds of the Sprint event also did not differ (**Table 2**). Skiers changed sub-technique an average of 16 ± 2 times (14.4 per km) during each of the Sprint rounds and 192 ± 23 times (18.4 per km) during the Distance race.

**Table 2:** Overall mean velocities and finishing times for the entire course Sprint and Distance races.

	Distance (10.5-km)		Sprint (1.1-km)	

		Time-Trial	Semi-Final	Final
Velocity (m⋅s^-1^) [min–max]	5.5 ± 0.4 [4.7–5.7]	5.7 ± 0.2 [5.4–6.0]	5.7 ± 0.1 [5.5–5.9]	5.7 ± 0.2 [5.5–5.9]
Finishing time (s) [min–max]	1926 ± 125 [1860–2180]	195 ± 9 [188–210]	196 ± 4 [192–202]	195 ± 7 [189–206]

By distance, DP was utilized to the greatest extent for both the 1.1-km Sprint rounds and the 10.5-km event, followed by Tuck, DS and Turn, with KDP being employed least and only by three participants during the Sprint (**Table 3**).

**Table 3:** Velocities, cycle lengths and cycle rates, and usage by distance and time (mean ± SD), with the various sub-techniques for all three Sprint rounds (SP) and the 10.5-km Distance event (DI).

	Velocity (m⋅s^-1^)		Cycle length (m)		Cycle rate (cycle⋅min^-1^)		Usage by distance (%)		Usage by time (%)	
Technique	SP	DI	SP	DI	SP	DI	SP	DI	SP	DI
DP	6.1 ± 0.2^∗∗^	5.5 ± 0.3	5.3 ± 0.4^∗∗^	5.7 ± 0.3	69.6 ± 4.2^∗∗^	59.1 ± 4.1	54 ± 3^∗∗^	49 ± 4	51 ± 4	48 ± 4
DS	3.2 ± 0.2ˆ	3.0 ± 0.2	2.5 ± 0.1^∗∗^	2.8 ± 0.1	80.6 ± 2.8^∗∗^	68.9 ± 3.4	13 ± 1^∗^	10 ± 2	22 ± 2^∗^	18 ± 4
KDP^#^	4.5 ± 0.2	4.2 ± 0.2	5.3 ± 0.4	5.2 ± 0.1	50.7 ± 3.1	48.7 ± 1.6	1 ± 2^∗∗^	4 ± 2	1 ± 3^∗∗^	5 ± 2
Tuck	9.1 ± 0.3^∗∗^	8.8 ± 0.1	–	–	–	–	14 ± 3^∗∗^	20 ± 2	9 ± 2^∗∗^	12 ± 1
Turn	7.8 ± 0.5^∗∗^	5.7 ± 0.4	–	–	–	*–*	9 ± 2^∗^	8 ± 0.3	6 ± 2^∗∗^	7 ± 0.3

*^∗∗^p = 0.03 compared to the other event, ^∗^p = 0.06 compared to the other event, ˆp = 0.09 compared to the other event, ^#^KDP was used by only 3 participants in the 1.1-km event.*

Macro-kinematic variables for each round of the Sprint finals (not presented) were similar to the Sprint qualification round. In all cases, the velocity was fastest when using the Tuck sub-technique, followed by Turn, DP, KDP, and DS, in that order (**Table 3**). The mean velocities with Tuck, Turn, DP, and DS were significantly higher for the Sprint, with no difference for KDP. During the Sprint the DP and DS cycle rates were significantly higher, and the DP and DS cycle lengths significantly lower, compared to the Distance event, with similar values in each event observed for KDP.

### Common Terrain

The mean velocities achieved by the skiers on the common terrain during the second and third laps of the Distance race were 5.3 ± 0.4 m s^-1^ (range 4.5–5.5) and 5.3 ± 0.5 m s^-1^ (range 4.4–5.8) respectively. In comparison, the overall velocities for the Sprint qualification, semi-final and final rounds were 5.8 ± 0.2 m s^-1^ (range 5.5–6.1), 5.8 ± 0.1 m s^-1^ (range 5.6–5.9) and 5.8 ± 0.2 m s^-1^ (range 5.5–6.0) respectively. Interestingly, the range in this velocity was narrower during the Sprint semi-final. On the common terrain there were sub-technique transitions 14 ± 2 times during the Sprint rounds and 15 ± 2 times during the Distance laps.

**FIGURE 2:**
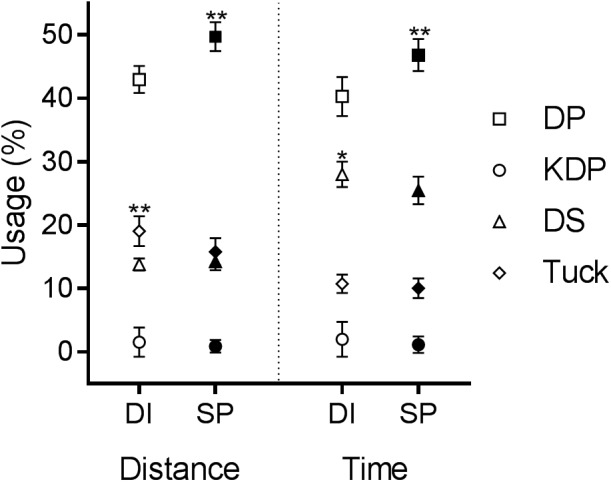
Sub-technique usage (%) in terms of distance and time on the 1.0 km of common terrain (*n* = 6). Open symbols, Distance; closed symbols, Sprint. ^∗∗^*p* = 0.03 compared to the other event, ^∗^*p* = 0.09 compared to the other event.

When on common terrain the sub-technique DP was utilized to the greatest extent, followed by Tuck, Turn (not presented) and DS (**Figure 2**), with KDP being employed least and only by three participants during the Sprint. The percentage of the total distance covered using DP was greatest in the Sprint (SP 50% v DI 43%, *p* = 0.03, MDiff = 15%), with a similar drop in the proportion of total time (SP 47% v DI 40%, *p* = 0.03, MDiff = 15%). With DS, the % distance was similar for both events, but percentage time was lower during the Sprint event as a consequence of the higher velocity (SP 25% v DI 28%, *p* = 0.09, MDiff = -10%). The time spent using Tuck was similar for both Sprint and Distance races, with slightly more rapid mean Distance velocity resulting in a longer distance (SP 16% v DI 19%, *p* = 0.03, MDiff = -19%). Mean KDP in usage was similar for both time and distance during both events. In terms of distance, unclassified techniques (Misc) were employed during 10 ± 3% of the Sprint event and 14 ± 2% of the Distance event. Regarding the Misc category, 3% of this in Sprint and 4% in Distance were attributed to transitions between sub-techniques, while 4% in Sprint and 6% in Distance were irregularities associated with Turns (i.e., where the skier had stopped performing a specified technique without yet beginning to change direction or had finished changing direction but not yet begun skiing with a specified technique again.

**FIGURE 3:**
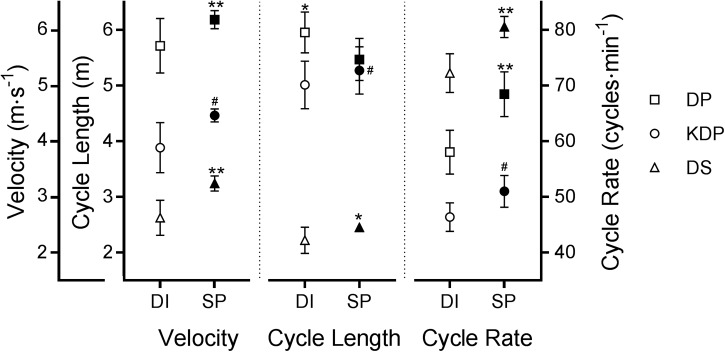
Mean velocities, cycle lengths and cycle rates (±SD) with the various sub-techniques on the 1.0-km of common terrain. Open symbols, Distance; closed symbols, Sprint; DP, double pole; KDP, kick-double pole; DS, diagonal stride. Note that KDP was used by only 3 participants in the Sprint event. ^∗∗^*p* = 0.03 in comparison to the other event, ^∗^*p* = 0.06 in comparison to the other event. ^#^Higher mean value but no statistical significance since only 3 Sprint skiers used KDP.

Sub-technique velocities on the common terrain exhibited the same relative rank as for the entire course (**Figure 3**). During the Sprint the mean velocities for DP (6.2 ± 0.2 v 5.7 ± 0.5 m s^-1^, *p* = 0.03, MDiff = 8.2%) and DS (3.2 ± 0.2 v 2.6 ± 0.3 m s^-1^, *p* = 0.03, MDiff = 22%) were higher (**Figure 2** – left panel). Although KDP was employed by only three athletes during the Sprint, for all three the velocity with this sub-technique was higher than the average for the Distance event (4.5 ± 0.2 v 3.9 ± 0.5 m s^-1^, *p* = 0.25 MDiff = 12%). Tuck velocity was slightly lower overall during the Sprint (9.1 ± 0.3 v 9.5 ± 0.3 m s^-1^ *p* = 0.03, MDiff = -4%). In contrast to observations on the entire course, the mean velocity for Turn on the common terrain was similar for both events. Minimum and maximum velocities for each of the sub-techniques are presented in **Table 4**.

**Table 4:** Mean, minimum and maximum velocities (±SD) for the various sub-techniques on the 1.0-km of common terrain.

	Distance Velocity (m⋅s^-1^)			Sprint Velocity (m⋅s^-1^)		
Technique	Mean	Min	Max	Mean	Min	Max
Tuck	9.3 ± 0.5	9.0 ± 0.8	9.5 ± 0.3	8.9 ± 0.6^∗^	8.6 ± 1.1	9.3 ± 0.2
DP	5.7 ± 0.5	3.6 ± 0.7	8.2 ± 0.6	6.2 ± 0.2^∗^	4.2 ± 0.4	8.6 ± 0.4
KDP^#^	3.9 ± 0.5	3.6 ± 0.4	4.3 ± 0.7	4.5 ± 0.2^∗^	4.2 ± 0.4	4.7 ± 0.4
DS	2.6 ± 0.3	1.7 ± 0.3	4.0 ± 0.3	3.2 ± 0.2^∗^	2.1 ± 0.2	4.6 ± 0.6

*DP, double pole; KDP, kick-double pole; DS, diagonal stride. ^∗^p = 0.03 compared to the other event, ^#^KDP was used by only 3 participants in the 1.1-km event.*

During the Sprint, mean cycle lengths were shorter with DP (5.5 ± 0.4 v 6.0 ± 0.4 m, *p* = 0.06, MDiff = -9%), but longer for DS (2.5 ± 0.1 v 2.2 ± 0.2 m, *p* = 0.06, MDiff = 10%) and KDP (5.3 ± 0.44 v 5.0 ± 0.4 m, *p* = 0.25, MDiff = 5%) (**Figure 2** – center panel).

All sub-technique mean cycle rates were higher in the Sprint (DP 68.5 ± 4.7 v 58.0 ± 4.2 cycles/min, *p* = 0.03, MDiff = 16%; DS 80.6 ± 2.8 v 72.3 ± 3.6 cycles/min, *p* = 0.03, MDiff = 11%; KDP 50.7 ± 3.1 v 46.9 ± 3.1 cycles/min, *p* = 0.5, MDiff = 8%) (**Figure 2** – right panel).

## Discussion

### Overview

This is the first study of the macro-kinematics of elite female athletes during an entire on-snow competition, and also the first comparison of macro-kinematic parameters between Sprint and Distance cross-country skiing events. In terms of distance, DP was the sub-technique used most extensively in both events, followed by Tuck, DS, Turn, and KDP. KDP was employed relatively little, and during the Sprint event by only half the participants. When events were compared over common terrain we observed that mean race velocities were higher in the Sprint. Mean sub-technique velocities with KDP and DS on the common terrain were higher in the Sprint due to faster cycle rates and longer cycle lengths, while the DP velocity was higher despite a shorter cycle length. During the Sprint the percentage of total distance covered with DP was greater, with the use of Tuck lower and the percentage of both KDP and DS similar relative to the Distance event.

### Common Terrain Macro-Kinematics

On the common terrain both the overall velocity and velocities with DP, KDP, and DS were expected to be higher in the shorter Sprint event. Corresponding elevations in cycle rates were also expected, since on-snow correlations between higher cycle rates and higher velocities for all three of these cyclical classical sub-techniques were reported by Nilsson et al. (2004). While similar correlations have been observed by numerous other investigations, including the study on DP by Lindinger et al. (2009), on KDP with roller-skiing by Göpfert et al. (2013), and on DS on-snow by Andersson et al. (2014), this study confirmed these findings for all sub-techniques throughout an entire on-snow competition. The hypothesis proposed by Zory et al. (2005) to explain this relationship is that a high cycle rate minimizes the decrease in velocity during glide and recovery phases while concurrently reducing the duration of these two phases. Millet et al. (1998) reported that a higher cycle rate would come at a higher metabolic cost, but Zory et al. (2005) noted that this would not be a limiting factor in a Sprint event.

We speculated that the shorter mean DP cycle lengths in the Sprint could be due to usage of DP on steeper inclines before transition to KDP or DS. However, the similar usage of DS in terms of distance, as well as closer examination of where sub-techniques were used around the course, indicated that this was not the case. On sections where DP was used for both events, higher cycle rate in combination with shorter cycles were clearly used to generate the higher DP velocity in the Sprint. This decrease in cycle length with increasing velocity was also observed by Nilsson et al. (2004) on-snow for all cyclical sub-techniques when speeds progressed from “fast” to “maximum,” but with DP the cycle lengths decreased earlier, when progressing from “medium” to “fast” velocities.

While this phenomena was also observed with maximal velocities with DS on-snow by Andersson et al. (2014), the velocities in these studies were collected over short sections which may not be indicative of an entire competition. In Nilsson et al.’s (2004) research, the maximal DS and KDP velocities of 6.2 and 6.1 m s^-1^ respectively were collected over 60 m of flat snow; while Andersson et al.’s (2014) DS velocity of 5.6 m s^-1^ was recorded over 50 m up a 7.5° incline. In both instances, the velocities far exceed both the mean and maximal DS and KDP velocities seen here. With other studies also reporting increases in both cycle length and cycle rate with increased velocity at sub-maximal workloads (Vähäsöyrinki et al., 2008; Göpfert et al., 2013), it seems likely then that the highest DS and KDP velocities reached during the Sprint in this study were sub-maximal. In contrast, our mean Sprint DP velocity was comparable to the maximal DP velocity in Nilsson et al.’s (2004) study, (6.2 v 6.3 m s^-1^).

The use of sub-maximal speeds in Sprint competition may reflect pacing, with athletes being unable to maintain maximal velocities over the 1.1-km course, and/or tactically holding back for critical parts of the course. Alternatively, our athletes may not have reached maximal velocity in KDP and DS because of velocity thresholds for sub-technique transitions (**Figure 1**). As athletes attain higher velocities using these two sub-techniques, it becomes possible to change to a faster sub-technique (from DS to KDP, from KDP to DP, and for some, directly from DS to DP). With DP, the velocity threshold for transition to the next fastest technique (Tuck) is too high to be attained on flat terrain, so skiers increase DP velocity by elevating cycle rate at the expense of cycle length. Regardless, this highlights the need for more analysis in the competition environment where sub-techniques are not pre-determined.

#### Sub-technique Selection

It is well known that incline also has an effect on sub-technique selection (Sandbakk et al., 2012a; Pellegrini et al., 2013; Ettema et al., 2017). As indicated in **Figure 2**, in terms of distance DS was utilized on the common terrain to a similar extent, approximately 14%, during both Sprint and Distance events. Furthermore, the GPS traces indicate that DS is generally being used on the same course sections in both cases, which would appear to support the conclusion of Ettema et al. (2017) that incline is the primary driver of technique choice. However, it is also possible that the velocity and gradient thresholds for technique transition are passed at the same time, i.e., velocity decreases as gradient rises. Unfortunately, the gradient profile in this present study was not sufficiently detailed to be able to comment further on the effect of gradient on sub-technique transitions. As the slowest sub-technique, the percentage usage of DS in terms of time is much greater (28%) in the Distance event, while due to the faster velocity in the Sprint is only used 25% of the time.

We have observed the low and variable use of KDP previously (Marsland et al., 2017); among the three athletes that used KDP in the Sprint, the average usage in terms of distance was just 2%. In the Distance event, five skiers used KDP over less than 1% of the distance, while the sixth used it for 6%. The mean minimum and maximum velocities in **Table 4** clearly reveal that the minimum DP velocity and the maximum DS velocity overlap, with the range of KDP velocities falling within those of the other two sub-techniques. Some skiers may feel they are more efficient when using one sub-technique compared to another and the choice appears to reflect personal preference.

While DP is the dominant technique during the Sprint, being used on average to cover 50% of the distance, it is also known that on Sprint courses with relatively little climb or, in particular, in fast conditions, skiers race without wax and use DP as their only cyclical technique (in addition to Tuck and Turn). While this happens more frequently in men’s classic Sprints [and sometimes with Distance races (FIS, 2015)], women have been known to DP races without wax as well. Interestingly, in the current case it appears that the increased usage of DP in the Sprint (7% more in terms of distance) reflects primarily less usage of Tuck (-3%) and Misc (-4%) sub-techniques. This lower use of Tuck in the Sprint appears to be due to athletes transitioning earlier to DP, particularly going into the finish straight. However, the more extensive usage of irregular technique in the Distance event remains unexplained. A proportion of Misc is made up of the transitions between sub-techniques, however, the number of transitions and Misc velocities in both events were found to be similar.

### Limitations

#### Influence of Topography

A key component of our study design was comparing skier macro-kinematics on common terrain under the same conditions. Our observations on the full 10.5-km event highlight the influence of terrain and the challenges involved in comparing between different courses, even when the conditions are similar. For example, the lower Tuck velocities on the remaining 3.5-km loop compared to the analyzed 1.0-km section indicate that the Sprint downhill sections were steeper, as supported by the homologation data (average downhill gradients of 9% during the Sprint race and 6% during the 3.5-km Distance loops). Furthermore, the slower velocities and shorter and more rapid cycle lengths when utilizing DS on the Sprint course are consistent with steeper inclines (average uphill gradients of 12% during the Sprint versus 10% for the 3.5-km loop). A similar observation concerning the relationship between gradient and macro-kinematics while performing DS on rollerskis was reported earlier by Sandbakk et al. (2012a).

Considering technique usage, DP was utilized to a larger extent on the full 10.5-km course (49% of the distance compared to 43% on the 1.0-km section), while the slower DS was employed less extensively (10% compared to 14%). In general, coaches experience that a course with more moderate gradients on uphills promotes greater proportional usage of DP and less DS (as seen here), and, consequently, a higher mean velocity. The outcomes observed here provide a suitable explanation for why the 10.5-km and 1.1-km events had similar overall mean velocities.

Accordingly, care must be taken when comparing macro-kinematics from different courses. For example, the mean overall velocity for the 10.5-km event observed here (5.5 m s^-1^) was similar to the 5.4 m s^-1^ we observed in an earlier men’s classic 10-km competition (Marsland et al., 2017). Although the sub-technique velocities in this previous investigation (DP 5.7 m s^-1^, DS 3.4 m s^-1^, KDP 4.4 m s^-1^) were similar to the current study, in the earlier work these velocities were achieved utilizing longer cycle lengths and slower cycle rates. To what extent this difference can be attributed to gender, course topography, snow speed and/or other factors is unknown.

It is worth noting that different macro-kinematic combinations by our skiers were successful. Similar sub-technique velocities were achieved using different proportions of higher cycle lengths and lower cycle rates and vice versa. With our small participant numbers, no macro-kinematic trends could be associated with faster or slower skiers, however, it seems likely that different strategies may be better suited to the strengths and weaknesses of the individual skier.

### Implications and Future Directions

For coaches and athletes there are three main practical applications that are confirmed from this study. First, the macro-kinematic strategies when training for Sprint and Distance events should not be the same. Clearly, the ability to attain higher cycle rates across all sub-techniques is essential for Sprint performance. Secondly, the demands of competition with respect to the different sub-techniques depend to a great extent on the terrain, with different courses requiring a different emphasis. Finally, evaluation of the macro-kinematic characteristics of an individual athlete during both training and competition can provide information concerning relative strengths and weaknesses that can help improve performance. Future studies in this area, involving more participants, should examine macro-kinematic trends of the best athletes in different events, at the same time considering variations in this respect during an event. In addition, assessment of potential gender-related differences over entire courses should provide valuable novel insights.

## Conclusion

Cross-country skiers can increase velocity by elevating cadence, increasing power (reflected in longer cycle lengths), and/or changing to a faster sub-technique. By monitoring macro-kinematics continuously throughout Sprint and Distance competitions on the same terrain we were able here to examine how these three mechanisms interact. Differences in the macro-kinematic characteristics and strategies utilized between Sprint and Distance events were confirmed, while at the same time the challenges of comparing between courses with different topographies and evaluating different factors influencing sub-technique selection were highlighted. Further insights are likely to be gained from examining differences in the macro-kinematic strategies of individuals within each event, and by continuing to analyze additional in-competition data.

## Author Contributions

FM, JA, GW, and DC: designed the study. FM: collected, processed, and analyzed the data. FM, JA, GW, H-CH, and DC: interpreted the results and wrote the paper.

## Conflict of Interest Statement

The authors declare that the research was conducted in the absence of any commercial or financial relationships that could be construed as a potential conflict of interest.
